# Role of expectations in clinical outcomes after deep brain stimulation in patients with Parkinson’s disease: a systematic review

**DOI:** 10.1007/s00415-023-11898-6

**Published:** 2023-07-30

**Authors:** Francesca Mameli, Eleonora Zirone, Roberta Girlando, Elena Scagliotti, Giulia Rigamonti, Edoardo Nicolò Aiello, Barbara Poletti, Roberta Ferrucci, Nicola Ticozzi, Vincenzo Silani, Marco Locatelli, Sergio Barbieri, Fabiana Ruggiero

**Affiliations:** 1 Fondazione IRCCS Ca’ Granda Ospedale Maggiore Policlinico, Via F. Sforza, 35, 20122 Milan, Italy; 2 ASST Santi Paolo e Carlo, San Paolo University Hospital, Milan, Italy; 3 Department of Neurology and Laboratory of Neuroscience, IRCCS Istituto Auxologico Italiano, Milan, Italy; 4 Department of Oncology and Hemato-Oncology, Università degli Studi di Milano, Milan, Italy; 5 Department of Pathophysiology and Transplantation, “Dino Ferrari” Center, Università degli Studi di Milano, Milan, Italy

**Keywords:** Deep brain stimulation, Parkinson disease, Subthalamic nucleus, Patients’ expectations, Placebo

## Abstract

Deep brain stimulation (DBS) is a well-established treatment that significantly improves the motor symptoms of patients with Parkinson’s disease (PD); however, patients may experience post-operative psychological distress and social maladjustments. This phenomenon has been shown to be related to patients’ pre-operative cognitive representations, such as expectations. In this systematic review, we discuss the findings on the role of the expectations of patients with PD regarding the clinical outcomes of DBS to identify areas of intervention to improve pre-operative patient education and promote successful post-operative psychosocial adjustment. PubMed was searched for relevant articles published up to 16 January 2023. Of the 84 identified records, 10 articles focusing on the treatment expectations of patients with PD undergoing DBS were included in this review. The selected studies were conducted among cohorts of patients with different DBS targets, among which the most common was the bilateral subthalamic nucleus. Overall, the data showed that patients’ expectations contribute to treatment efficacy. Experiments investigating the placebo effect itself have shown clinical improvement after the induction of positive therapeutic expectations; conversely, unrealistic treatment expectations can affect patient satisfaction after surgery, clinical outcomes, and subjective well-being. This review highlights the need for routine clinical practice to better investigate and manage patients' pre-operative expectations, as well as multidisciplinary education to improve patient satisfaction and psychosocial adjustment after DBS.

## Introduction

Parkinson’s disease (PD) is a progressive neurodegenerative disorder characterised by a variety of motor and non-motor impairments primarily caused by the selective loss of dopaminergic neurones in the nigrostriatal pathway [1]. Bilateral deep brain stimulation (DBS) reduces motor symptoms and dopaminergic-related complications in patients with advanced PD [2–5]. While successful functional neurosurgery leading to the sudden alleviation of symptoms is expected to significantly improve patients’ quality of life (QoL), growing evidence suggests that such positive effects are questionable [6–11]. This phenomenon, first characterised by Bladin as the ‘*Burden of Normality*’, has been mostly investigated in patients with medically intractable epilepsy undergoing anterior temporal lobectomy [12, 13]; despite successful treatment and alleviation of seizures, some patients experienced psychosocial maladjustments (e.g., difficulties in discarding sick role behaviours, restructuring body image and identity, and readjusting social relationships in personal and professional contexts).

Previous studies have suggested that pre-operative patient expectations, defined as future-directed beliefs about the occurrence of a specific outcome [14], play a key role in the post-operative psychosocial adjustment process [15–17]. Patients undergoing treatment create their own expectations based on several factors, including independently acquired information, communication with specialists, the chronicity and pervasiveness of their condition, hopes and fears, and aspects of their personality [14, 18–20]. Moreover, they often obtain information from sources that describe the best possible treatment results, which can create unrealistic expectations that may interfere with the therapeutic benefits of DBS [19].

A fundamental assumption of the QoL literature is that the physical, psychological, and social domains of health are greatly influenced by beliefs, expectations, and perceptions [21]. Theoretically, these beliefs translate objective health outcomes into the actual QoL experienced by patients. Therefore, effective measurement of outcomes should span the pre- to post-operative transition by examining the magnitude of disparity between pre-operative expectations and post-operative reality. Pre-operative expectations of the benefits of surgery may influence the perceived success of DBS, and in turn, the perception of post-operative QoL.

The relevance of patients’ expectations on treatment outcomes has been investigated in various medical contexts, such as cardiovascular diseases [22–24], tumours [17, 25, 26], chronic pain [27–29], and addictions [30]. Therefore, it is surprising that pre-operative expectations have not been thoroughly examined in previous studies that have assessed post-operative QoL in patients with PD undergoing DBS.

To identify areas of intervention capable of improving pre-surgery patient education and post-operative psychosocial adjustment, this review aimed to synthesise available studies that evaluated the therapeutic expectations of patients with PD undergoing DBS.

## Methods

The bibliographic search was conducted by entering the following query in PubMed: ((“Parkinson Disease” [MeSH Terms] OR “Parkinson Disease” [Title/Abstract] OR “Parkinson’s Disease” [Title/Abstract]) AND (“Deep Brain Stimulation” [MeSH Terms] OR “Deep Brain Stimulation” [Title/Abstract]) AND (“Patients Expectations” [Title/Abstract] OR “Patient Expectations” [Title/Abstract] OR “Patient Expectation” [Title/Abstract] OR “Expectation” [Title/Abstract] OR “Expectations” [Title/Abstract] OR “Preoperative Expectations” [Title/Abstract] OR “Expectancy” [Title/Abstract] OR “Treatment Expectations” [Title/Abstract])) AND (English [Filter]). The search yielded a total of 84 articles, update to January 16, 2023.

We included studies that met the following inclusion criteria: articles written in English; articles including data on patients with PD undergoing DBS; and studies that investigated the patients’ expectations of the treatment outcome through behavioural paradigms and/or standardised or ad hoc instruments (e.g., questionnaires, interviews, clinical scales). No restrictions were applied to the patients’ age, disease duration, disease severity, or the DBS target. We excluded articles that investigated the therapeutic expectations of patients with PD not undergoing DBS, studies that included data on patients with other diseases undergoing DBS, reviews, commentaries, points of view, qualitative studies, and handbooks.

**Fig. 1:**
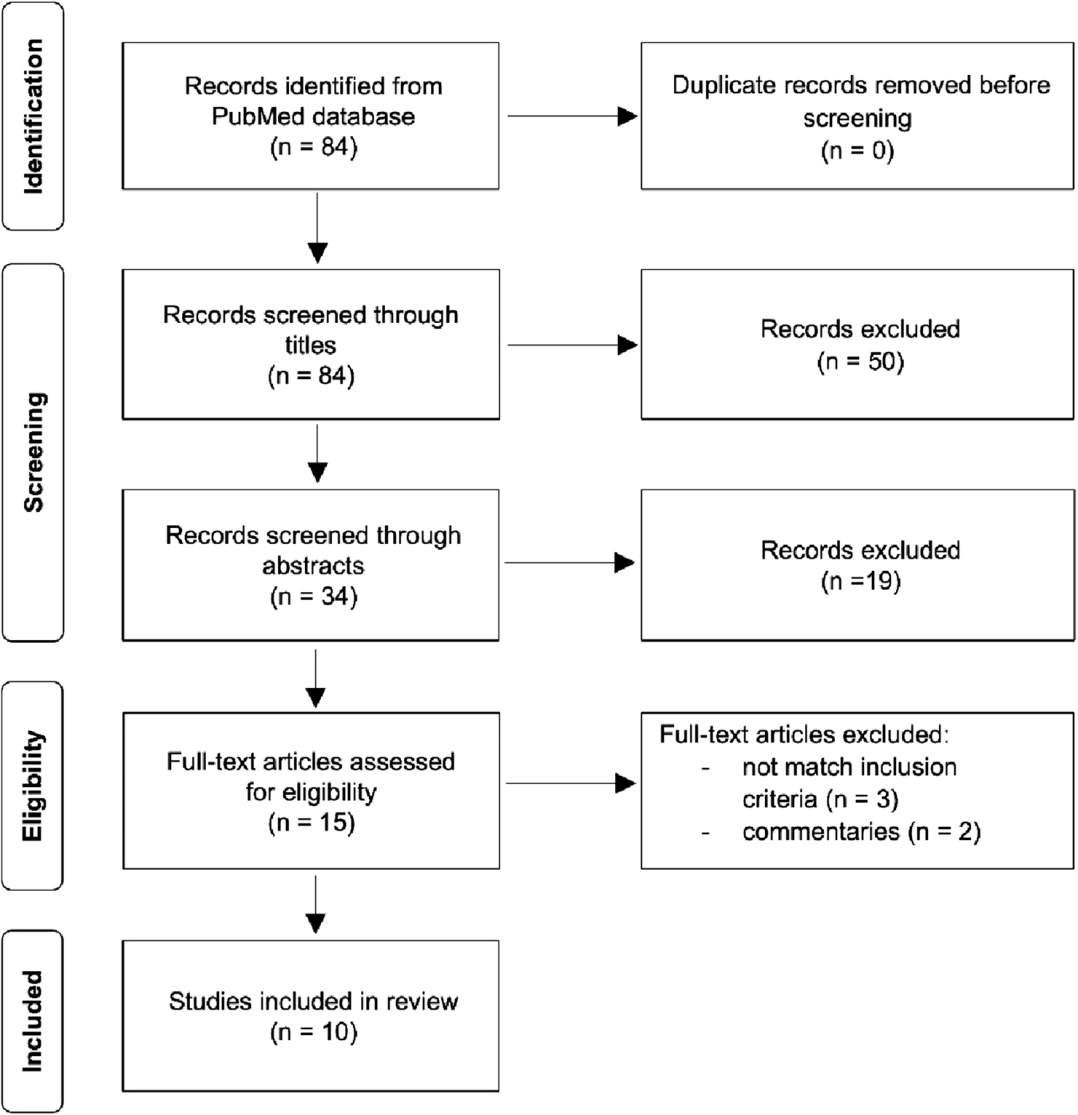
Flowchart of the study selection process

After checking for duplicates, two independent reviewers preliminarily selected articles based on the titles and abstracts. The remaining articles were further screened by examining the entire manuscript. Disagreements between reviewers were resolved by a third author. A flowchart of the systematic selection process is shown in Fig. 1.

## Results

Ten papers dealing with the role of patients’ therapeutic expectations on the clinical outcome of DBS were included (n = 272 PD patients). The extracted data are summarized in Table 1 and subdivided according to whether the relationship between therapeutic expectations and treatment outcomes was experimentally manipulated (Type 1; n = 4 studies, n = 65 patients) or assessed at an observational, ecological level (Type 2; n = 6 studies, n = 207 patients). All included studies used a within subject design; only one type 2 study used a mixed approach within and between subjects. In the vast majority of studies (80%), stimulation was bilaterally directed to the subthalamic nucleus (STN); the targets of the remaining studies also included the unilateral STN and the globus pallidus internus [37]. Two postoperative time points were included in three of four type 1 studies (75%) [31, 33, 34]. All type 1 studies addressed motor outcomes, while two (50%) studies addressed cognitive outcomes [33, 34]; none of these studies reported an impact of expectations on cognitive functioning, while three of them (75%) showed an expected association between positive/negative expectations and better/worse motor skills [31–33]. In type 2 studies, 5 of 6 studies (83.3%) measured both pre- and postoperative time points, while one study only measured patients' postoperative expectations [39]. In type 2 studies, expectancies were mostly represented by non-standardized questionnaires (66.7%), with only two studies using standardized instruments to record health-relate QoL [36, 39]. All but one type 2 studies (83.3%) focused explicitly on examining the relationship between patients’ expectations prior to surgery and their satisfaction with the outcome of the surgery itself. The results of the type 1 and type 2 studies are narratively summarized in the next two sections.

**Table 1:** Characteristics of reviewed studies exploring expectations of patients with PD undergoing DBS

Author and year	PD patients	Age (years)	PD duration (years)	DBS target	Study design	Data collection time point(s)	Experimental paradigm	Outcome measure(s) of expectations’ manipulation	Main results
Studies on manipulation of therapeutic expectations									
Pollo et al., 2002 [31]	7 (3 M, 4 F)	58.57 ± 8.62^a^	15 ± 6.19^a^	Bilateral STN	Within subject	T0: 15.57 ± 7.95^a^ months after surgery T1: one week after T0	Patient expectations’ manipulation conditions: Bad performance (B) Good performance (G) Manipulation of DBS stimulation intensity: T1 reduction to 20% T2 increase to 40% T3 increase to 100%	Motor function	Effect of expectations’ manipulation on hand velocity: patients were faster in G than in B condition (T1: p = 0.027; T2: p = 0.045)
Mercado et al., 2006 [32]	10 (8 M, 2 F)	61 (42–78)^b^	14 (6–23)^b^	Bilateral STN	Within subject	T0: after surgery (exact timing NR)	Patient expectations’ manipulation conditions: Aware vs Blind in StimON/StimOFF conditions	Motor function	Effect of expectations’ manipulation (p = 0.049): StimOFF: worse motor function in aware than blind condition; StimON: better motor function in aware than blind condition
Keitel et al., 2013[33]	24 (12 M, 12 F)	62.83 ± 1.90^a^	13.52 ± 6.24^a^	Bilateral STN	Within subject	T0: after surgery T1: one day after T0	Patient expectations’ manipulation conditions: Placebo Nocebo Control Pharmacological conditions: MedON MedOFF	Motor function; Cognitive function	Effect of expectations’ manipulation only on proximal movements in MedOFF: Higher speed in placebo than in nocebo condition (p = 0.005); No difference between placebo/nocebo and control condition (p = 0.090*, p = 0.240*)
Keitel et al., 2013 [34]	24 (19 M, 5 F)	64.17 ± 1.60^a^	11.58 ± 6.21^a^	Bilateral STN	Within subject	T0: after surgery T1: one day after T0	Patient expectations’ manipulation conditions: Placebo Nocebo Control Pharmacological conditions: MedON MedOFF	Motor function; Cognitive function	No effect of patients’ expectations manipulation on motor (all p > 0.390) and cognitive (all p > 0.120) function

Author and year	PD patients	Age (years)	PD duration (years)	DBS target	Study design	Data collection time point(s)	Expectation measure	Main results
Studies on the relationship between therapeutic expectations and perceived outcomes after surgery								
Maier et al., 2013[35]	30 (18 M, 12 F)	61.20 ± 8.65^a^	11.97 ± 6.79^a^	Bilateral STN	Within subject	T0: before surgery (exact timing NR) T1: 3 months after surgery	Semistructured interview	Patients with subjective negative DBS outcome were characterised by pre-surgery unrealistic expectations (mental state improvement, p = 0.028)
Hasegawa et al., 2014 [36]	19 (10 M, 9 F)	59.80 (SD NR)^a^	11 (range NR)^b^	Bilateral STN	Within subject	T0: before surgery (exact timing NR) T1: 6 months after surgery	Modified version of PDQ-39	Improved PDQ-39 scores (mobility, ADL, stigma, cognition, and bodily discomfort domains; but less than expected (p = 0.008.) Satisfaction correlated with fulfilment of expectations (p < 0.001) but not with quantitative changes in PDQ-39 scores
Knoop et al., 2017 [37]	29 (21 M, 8 F)	66.80 ± 10.80^a^	15.10 ± 8.59^a^	Bilateral STN (62.10%) Unilateral STN (17.40%) Bilateral GPi (13.80%) Unilateral VIM (6.90%)	Within subject	After surgery (exact timing NR)	Ad hoc questionnaire	Most cited symptoms for pursuing DBS: tremor (79.30%), dyskinesias (24.10%) and rigidity (13.80%) 71.40% of patients discussed expectations prior to surgery: 3.60% not adequately informed about DBS treatment; 17.90% only partially informed 100% of subjects were in some agreement that their expectations from DBS surgery were met
Karl et al., 2018 [38]	52 (31 M, 21 F)	66.10 ± 7.90^a^	19.70 ± 5.60^a^	Bilateral STN	Within subject	T0: before surgery (exact timing NR) T1: 8.20 ± 2.60^a^ years after surgery	Ad hoc questionnaire (only at T1)	No correlation between pre-surgery expectations and post-surgery satisfaction (p = 0.060)
Lin et al., 2019 [39]	15 (9 M, 6 F)	60.50 ± 6.40^a^	10 (5–15)^b^	Bilateral STN	Within subject	T0: before surgery (exact timing NR) T1: 6 months post surgery T2: 6 ± 1^a^ years post surgery	Modified PDQ-39	Pre-surgery patient expectations were higher than the PDQ-39 score both at T1 (p = 0.002) and T2 (p = 0.001) Positive correlation between satisfaction and patient expectations at T1 (p < 0.001), but not at T2 (p = 0.114)
Yen et al., 2021[40]	Exp.1: 62 App users (46 M, 16 F) Exp. 2: 14 App users + DBS (12 M, 2 F) 12 DBS control group (9 M, 3 F)	App users: 59.16 ± 9.11^a^ App users + DBS: 56.34 ± 11.94^a^ DBS control group: 62.25 ± 5.39^a^	App users: 8.60 ± 4.29^a^ App users + DBS: 8.54 ± 4.03^a^ DBS control group: 12.17 ± 4.15^a^	App users + DBS: Bilateral STN (6) Unilateral STN (3) Bilateral GPi (5) DBS control group: Bilateral STN (5) Unilateral STN (3) Bilateral GPi (2) Unilateral GPi (2)	Within subject Between subject	T0: before using the app (exact timing NR) T1: after using the app (exact timing NR) T2: before surgery (exact timing NR) T3: after surgery (exact timing NR)	Ad hoc questionnaire	DBS-Edmonton app improved DBS-related knowledge and patient satisfaction post-surgery (p = 0.014)

*ADL* activities of daily living, *DBS* Deep Brain Stimulation, *Exp* experiment, *F* female, *GPi* Globus pallidus pars interna, *M* male, *MedOFF* off antiparkinsonian medication, *MedON* on antiparkinsonian medication, *NR* not reported, *PDQ-39* 39-items Parkinson’s Disease Questionnaire, *StimON* stimulation on, *StimOFF* stimulation off, *STN* subthalamic nucleus, *T* time-point, *VIM* ventral intermediate nucleus of the thalamus

*Correct with Bonferroni significance level

^a^Data presented as the means with standard deviation (SD)

^b^Data presented as the means with range

### Manipulation of therapeutic expectations

To explore how opposite expectations modulate motor performance in patients with PD undergoing STN-DBS, Pollo et al. [31] modulated current intensity delivered through the neurostimulation implant in seven subjects while they executed a visual directional-choice task. First, the current was markedly reduced (phase 1), then slightly increased (phase 2), and finally restored to normal intensity (phase 3). The patients were subjected to two different conditions. In the ‘good performance condition’ (‘G condition’) the researchers tried to induce positive expectations about motor performance: in phase 1, the patients were told that nothing would change; in phase 2, they were told that there would be a big improvement of motor performance; and when the stimulation returned to the initial values, nothing was said. In the ‘bad performance condition’ (‘B condition’) patients were told the truth about what was going to happen: they were informed that motor performance was going to worsen, then that it was going to improve, and then it would finally return to normal. The velocity of right-hand movement was assessed using a movement analyser. After 30 min of decreased current intensity, participants in the ‘B condition’ were significantly slower than those in the ‘G condition’ (p = 0.027), who also showed a significantly better performance when the current was slightly increased (p = 0.045). These data show that movement velocity in patients undergoing DBS can be modulated by different verbally induced expectations about motor performance; when good motor performance is expected, hand movement is faster and vice versa. In addition, all these effects occurred within minutes, suggesting that expectations induce neural changes very quickly. Their results demonstrated that placebo-induced expectations influence treatment outcomes in patients with PD undergoing DBS.

Mercado et al. [32] aimed to determine whether the degree to which a patient with PD expects therapeutic benefits influences the magnitude of the improved motor response in 10 patients with idiopathic PD treated with bilateral STN-DBS. After 12 h of stimulation interruption and no antiparkinsonian medications, the motor functions of patients were evaluated using the Unified Parkinson’s Disease Rating Scale-part III (UPDRS-III) in each of the following experimental conditions: the patient was aware that the stimulator was ON or OFF, and the patient was blinded to the stimulator being ON or OFF. The results of the UPDRS-III showed a better clinical score in the ON vs. OFF condition (p = 0.0001), confirming the efficacy of DBS in managing motor symptomatology. Interestingly, when the stimulation was OFF, patients aware of this condition had worse UPDRS-III motor scores than when they were blinded to this condition (mean ± SD: 50.70 ± 16.60 vs. 47.60 ± 12.20). Conversely, when the stimulation was ON, the UPDRS-III motor scores were better when the patients were aware of the stimulation compared with when they were blinded (mean ± SD: 30.60 ± 13.05 vs. 34.50 ± 13.20; p = 0.049). No significant differences emerged among the experimental conditions for the tremor (p = 0.230) and rigidity (p = 0.100) sub-scores, whereas the differences in bradykinesia tended to be significant (p = 0.059). This study showed that the expectation of positive or negative therapeutic benefits of DBS can modulate motor outcomes in opposing directions, as measured by standardised motor scales.

To analyse the role of expectations on non-motor symptoms, Keitel et al. [33] verbally induced three different expectancy conditions in 24 patients with PD with and without antiparkinsonian drugs (MedON, MedOFF). Positive expectations were induced by telling the patients that STN-DBS would be activated with parameters that would strongly improve motor function (placebo condition). Negative expectations were induced by informing the patients that the stimulator would be activated with parameters that would strongly impair motor function (nocebo condition). Finally, neutral expectations were induced by indicating that the parameters would not have an impact on motor function (control condition). Immediately after the expectations were verbally induced, the patients were asked to rate the extent of the expected improvement, impairment, or steadiness of their motor state on a numerical rating scale (NRS). Motor function was assessed using the Movement Disorder Society-Sponsored Revision of the UPDRS-III (MDS-UPDRS III) and quantitative kinematic analysis of proximal and distal movement tasks, whereas cognitive functions were tested using four verbal fluency tests: a formal lexical test, a semantic category test, a formal lexical category change test, and a semantic category change test. Moreover, to identify potential mediators of placebo and nocebo responses, patients’ state, and trait anxiety (State-Trait Anxiety Inventory—STAI-S; STAI-T) and beliefs about medicines (Beliefs about Medicines Questionnaire—BMQ) were evaluated using questionnaires. In the MedOFF condition, proximal but not distal movements were modulated by expectations, but only when comparing the placebo and nocebo conditions was the difference in the mean angular speed of the proximal task significant. Patients showing an improvement in motor function in the proximal task of at least 25% compared with that in the control condition were classified as placebo responders (10/24 in MedOFF), and those with an impairment ≥ 25% compared with the motor function in the control condition were considered nocebo responders (1/24 in MedOFF). No global effect of expectation on verbal fluency tasks was found; however, in the placebo responders’ subgroup, a trend of impaired lexical verbal fluency, namely, producing fewer words, in the placebo condition compared with that in the control condition was highlighted (mean ± SD; placebo: 9.10 ± 1.84 vs. control: 10.40 ± 1.45; p = 0.080). The responders and non-responders did not differ significantly in terms of anxiety, beliefs about medicine, or expectation ratings. Altogether, these data suggest that positive motor expectations could exert motor placebo and cognitive nocebo responses, indicating that the effects of expectations on STN-DBS closely resemble the actual effects of STN-DBS, with an improvement in proximal movements and impairment in lexical verbal fluency.

To extend their previous results, Keitel et al. [34] applied the same paradigm described above to assess the effects of expectations (positive, negative, neutral) on 24 tremor-dominant patients with PD undergoing STN-DBS in MedON and MedOFF conditions. In addition to the assessments described above (NRS, MDS-UPDRS III, proximal and distal tasks, STAI-S, STAI-T, BMQ, and verbal fluency tasks), resting tremors were evaluated using a motion detection system. In this study, placebo and nocebo responders were defined as those who demonstrated ≥ 10% improvement (placebo) or worsening (nocebo) of their resting tremor compared with that in the control condition. At the group level, expectations did not modulate the resting tremors or bradykinesia of proximal and distal movements in the MedON and MedOFF conditions (all p > 0.390). Nocebo responders in the MedON condition were characterised by impairment in semantic verbal fluency (p < 0.050), but the sample size was limited (5/20), and no effect was observed in placebo responders (8/20 in MedON; all p > 0.120). As in their previous study, the responders and non-responders did not differ significantly in terms of anxiety, beliefs about medicines, or expectation ratings. The absence of any significant modulation at the group level combined with the small number of placebo and/or nocebo responders did not allow us to draw strong clinically relevant conclusions from this study.

### Relationship between therapeutic expectations and perceived outcomes after surgery

To investigate patients’ subjective expectations and the impact of DBS on motor, emotional, social, behavioural, and cognitive functioning, as well as on activities of daily living and QoL, Maier et al. [35] conducted a semi-structured interview before and three months after surgery. Thirty participants were classified into three groups based on their subjectively reported outcomes: negative (n = 8), mixed (n = 8), and positive (n = 14). Clinical scales were administered to obtain quantitative measures of motor symptoms in both the MedON and MedOFF conditions (UPDRS-III), QoL (Parkinson Disease Questionnaire-39—PDQ-39), cognitive profile (short-term memory, verbal fluency, and attention domains), and neuropsychiatric status (apathy, depression, mania, and anxiety symptoms). Their analysis showed improvements in motor function and QoL after DBS (both p < 0.001). Regarding expectations, all patients expected an improvement in motor symptoms, but only the negative group more frequently indicated a pre-operative expectation of improvement of mental state (p = 0.028), a symptom that cannot be directly modified by DBS. Interestingly, three months after surgery, this group reported less perceived improvement in motor symptoms (p = 0.010), autonomy (p = 0.046), and QoL (p < 0.001), as well as worsening of their mental state (p = 0.002). In addition, the negative group had higher scores for apathy and depression in both the pre- and post-operative phases (all p < 0.005) and a worse PDQ-39 score post-surgically (p < 0.001). The authors identified apathy and depression as significant predictors of negative subjective outcomes; therefore, optimal cut-off scores were suggested (36/37 for the Apathy Evaluation Scale; 16/17 for the Beck Depression Inventory II). These findings show that having unrealistic expectations and mood disturbances during screening can lead to disappointment with DBS outcomes.

Hasegawa et al. [36] tested 19 subjects before and six months after surgery. They assessed health-related QoL using a modified version of the PDQ-39, which also investigated the patients’ expectations of improvement in the following domains: mobility, activities of daily living, emotional well-being, stigma, social support, cognition, communication, and bodily discomfort. In addition, the patients completed a locally developed questionnaire to assess their personal experiences and overall satisfaction with DBS. Their analysis showed that patients expected a significant improvement after DBS in all investigated domains. Six months after surgery, the patients showed significant improvement in the physical but not psychosocial domains (social support, communication, and cognition); however, they expected even greater improvements, as shown by the substantial disparity between the expected and actual changes (median [interquartile]: 24.0 [15.0] vs. 14.0 [22.5]; p = 0.008). However, the satisfaction questionnaire revealed that most patients felt that the surgery fulfilled their expectations. Moreover, satisfaction correlated with the fulfilment of expectations (r = 0.910, p < 0.001) but not with quantitative changes in PDQ-39 scores (all p > 0.05). According to the authors, a possible explanation for why psychosocial domains failed to improve after intervention could be that both DBS and the best medical therapy do not significantly alter the neurophysiological basis of the psychosocial factors represented in the PDQ-39.

To investigate whether patients’ expectations regarding DBS were met post-operatively and to identify gaps in their pre-operative education, Knoop et al. [37] conducted a retrospective study of 29 patients with PD. A non-standardized 27-item questionnaire was developed to evaluate patient expectations, pre-operative education, and overall satisfaction with DBS using Likert scales and free-response questions. The results showed that most patients reported receiving adequate information regarding the limitations of DBS. The most commonly reported symptoms that caused patients to pursue DBS were those that had better chances to improve (i.e. tremor, dyskinesias and rigidity), whereas symptoms that did not necessarily improve after surgery (i.e. walking and balance problems, freezing of gait, and impaired handwriting) were cited only in few cases, and non-motor symptoms were rarely mentioned. Moreover, only one patient (3.6%) reported not being adequately informed about DBS, while 17.9% felt that they were only partially informed. Most participants (71.4%) reported having been asked about their expectations of DBS before surgery; however, this was documented in the medical charts in only 48.3% of cases. All patients indicated that the benefits of DBS met their expectations, at least partially, but only 46.4% strongly agreed. Expectations of improvements in motor symptoms and medication reduction were mostly met, whereas expectations regarding non-motor symptoms (speech, balance, and walking problems) were not. Despite the overall satisfaction among this sample, the authors highlighted the need to optimise the educational process for DBS surgery, specifically by introducing a standardised protocol and involving a multidisciplinary team. Indeed, due to the partial lack of medical charts on this aspect, the evaluation of patients’ expectations was carried out several years post-operatively, and this latency could have biased the results.

To assess satisfaction, expectations and overall, long-term, patient-centred outcomes, Karl et al. [38] evaluated 52 patients with PD after bilateral STN-DBS. Patients were assessed before and after surgery (mean ± SD: 8.2 ± 2.6 years) regarding their motor and non-motor symptoms (UPDRS I-IV), autonomy in daily life (Schwab and England scale), and QoL (PDQ-39). Moreover, a non-standardised, locally developed, DBS patient-centred outcome questionnaire was administered. This questionnaire was divided into two sections: the first focused on patients’ pre-operative expectations and their overall satisfaction with DBS treatment, whereas the second aimed to quantify the severity of their motor and non-motor symptoms before surgery and at the current time. All questions were scored on a ten-point scale, ranging from 0 (most ‘positive’ answer) to 10 (most ‘negative’ answer). Their results showed that most patients reported subjective improvements in dyskinesia, motor fluctuations, tremors, rigidity, and side effects of medication, whereas gait, balance, and non-motor symptoms were generally reported to be unchanged or worsened. Although pre-surgery counselling was conservative regarding the outcome that patients should expect, the pre-operative expectation target was set as very high (median score = 2); nevertheless, patients were highly satisfied with their DBS outcomes (median score = 1). While no significant relationship between expectations and satisfaction was found (r = 0.27, p = 0.060), a weak but significant correlation was observed between current satisfaction and patient-rated motor severity (r = 0.36, p = 0.010) and non-motor symptoms (r = 0.33, p = 0.020). Furthermore, worsening QoL (r = 0.57, p < 0.0001), insomnia (r = 0.43, p = 0.010), apathy (r = 0.40, p = 0.030), depression (r = 0.38, p = 0.010), pain (r = 0.37, p = 0.010), bradykinesia (r = 0.30, p = 0.030), and rigidity (r = 0.29, p = 0.040) were associated with decreased patient satisfaction. However, the authors highlighted two major limitations in their study design. First, expectations about DBS outcomes retrospectively evaluated do not necessarily match the actual pre-operative expectations. Second, the lack of UPDRS-III assessment at the time of the questionnaire did not allow a quantitative analysis of the impact of expectations on motor improvement.

To extend the preliminary findings of Hasegawa et al. [36], Lin et al. [39] explored expectations, satisfaction, and outcomes six years after STN-DBS surgery and compared them with those collected before and six months after surgery. Six years after surgery, 15 of the 19 patients from the original cohort completed a modified version of the PDQ-39 to assess their actual QoL and expected changes, as well as a satisfaction scale for DBS. Despite the initial improvement observed six months after surgery, the patients’ QoL (assessed with PDQ-39 sum and sub-scores) returned to pre-surgical levels by the 6-year follow-up, except for the improvement in the stigma domain (p = 0.011). Regarding expectations, the patients’ pre-operative expectations were higher than the actual post-surgical outcomes at both 6 months (p = 0.002) and 6 years (p = 0.001). However, no significant differences emerged between the expected and actual QoL at the two post-operative time points. Patients remained highly satisfied with the surgical outcome at the 6-year follow-up (mean satisfaction score = 83%), but the positive correlation between the fulfilment of expectations and the level of satisfaction observed 6 months after surgery (r^2^ = 0.92, p < 0.001) was no longer significant (r^2^ = 0.18, p = 0.114). The most satisfied patients (n = 9) indicated less favourable expected conditions at the 6-year follow-up compared with their pre-surgical expectations (p = 0.01), so they lowered their expectations over time; this change was not observed in the less satisfied group (n = 3; p = 0.890). These data suggest post-operative alignment between patients’ expectations and effective clinical outcomes.

Finally, Yen et al. [40] developed and assessed the DBS-Edmonton App, an educational tool explaining which PD symptoms are best managed by DBS treatment, in candidates for implants. They surveyed 62 patients before and after consulting the app regarding their knowledge and expectations of symptom control with DBS. Except for tremors and dyskinesia (for which volunteers demonstrated an excellent understanding at baseline), knowledge about the responsiveness of PD symptoms to DBS improved after using the DBS-Edmonton app; however, the difference was significant only for gait impairment. Most participants found the app informative, used it to assist in decision-making, and recommended it to other patients. Additionally, 14 of the 62 patients underwent DBS implantation, and to investigate whether the app consultation could enhance DBS satisfaction and outcomes, their clinical improvements were compared with those of 12 patients with PD who underwent DBS without using the app. All 26 patients were evaluated before and after surgery for motor (UPDRS-III) and non-motor symptoms (Non-Motor Symptoms Questionnaire), both off and on antiparkinsonian medication, as well as for goal attainment, improvements in functional status, and satisfaction with DBS. Between the two groups, no significant difference was found in the improvement of motor (p = 0.120) and non-motor symptoms (p = 0.660). Most patients were satisfied with DBS and functional improvement; however, those who used the app demonstrated a higher degree of satisfaction than those in the control group (p = 0.014). Although these results support the hypothesis that managing patient expectations before DBS is crucial to improving patient satisfaction by setting realistic expectations for symptom control, the differences between the experimental and control groups limit the interpretation of these results. The control group had significantly higher age (p = 0.020), disease duration, and non-motor symptom severity before surgery (p = 0.020) than those in the experimental group, hindering the generalisation of these results. Moreover, the mean time between DBS and the survey varied considerably between the two groups (app users: 6 months, control: 21.6 months), which was reported by the authors as a possible cause of the varying satisfaction levels of the two groups.

## Discussion

This study aimed to critically review the literature on the therapeutic expectations of patients with PD undergoing DBS. Despite their heterogeneities, the studies included in this review showed that the effects of DBS can be amplified by patients’ positive expectations (placebo effect) or, conversely, reduced by negative expectations (nocebo effect) [31–34].

Experiments examining the placebo effect itself have shown clinical improvement after positive treatment expectations have been elicited. Patients with DBS-STN improved their motor performance by 11.3% when they knew their stimulators were ON than when they were unaware of the stimulator state [32]. In addition, an interesting experiment, using an overtcovert experimental design, showed that positive expectation improves hand motor speed by about 60% [31]. This issue is particularly relevant in DBS candidates, given the high placebo response rate in the PD population (16%, range 0–55%), which increases with surgical interventions compared with pharmacological therapies [41]. Previous studies have shown that patients with PD represent an excellent model to study the placebo effect [1]. The administration of placebo drugs to these patients induces a significant release of endogenous dopamine in the striatum [42, 43], as well as alterations in neuronal firing patterns in the STN [44], which are associated with improved motor function. Therapeutic expectations have also been identified as one of the main psychological factors underlying placebo responses which trigger complex neurobiological phenomena [45, 46].

Interestingly, studies have highlighted that not all cardinal symptoms have the same susceptibility. The bradykinesia seems to be the only symptoms sensitive to verbal suggestions than tremors and rigidity [31–34]. Pollo and colleagues found a significant improvement of about 60% [31]; this trend was also confirmed in two other studies, which found an improvement between 9 and 16%, but not statistically significant [32, 33]. Indeed, evidence of the role of expectations in cognitive outcomes remains limited; only two studies found a partial effect on verbal fluency. The authors suggested that different phenotypes may respond differently to this placebo cognitive effect [33, 34].

No potential predictors were observed to characterise the placebo responders or non-responders. A comparison of the two groups showed no difference between the psychological and disease-related variables and expectation rating. Therefore, other factors may play a role in placebo response in patients with PD [33, 34]. Studies conducted on other pathologies observed that state and trait anxiety and dispositional optimism in analgesia and coping skills in irritable bowel syndrome mediate the placebo response [46]. Evidence of anxiety and depressive disorders suggests that genetic traits play a role in the occurrence of individual responses to placebos [47–49]. Identifying the predisposing factors for a placebo response in patients with PD would allow us to predict the patient’s responsiveness and improve the subject’s susceptibility, optimising the specific response to DBS.

Furthermore, post-operative satisfaction is often related to adequate pre-operative and post-operative expectations [35–40]. An analysis of the relationship between pre-DBS expectations and post-operative satisfaction has shown how overly optimistic expectations can negatively impact perceived outcomes and QoL in a short post-operative follow-up, despite an objective improvement in motor profile [35]. Perceptions of motor improvement in these patients were approximately 38% lower than those with adequate expectations. In addition, the impact of unrealistic expectations was also found on QoL and degree of autonomy, which were estimated to be about 88% and 52% lower, respectively [35]. The need for realistic patient expectations is emphasised because a discrepancy between the expected and actual effects of STN-DBS on the motor symptoms of PD may result in negative perceptions of the outcome and disappointment.

Longitudinal studies have shown how expectations can change over time: satisfied patients may lower their pre-operative expectations, aligning them with actual post-DBS outcomes. This change seems to allow patients to become satisfied with their surgical outcomes, even in the absence of objective improvements in the psychosocial domain [36], worsening of motor and non-motor symptoms [38], or worsening of QoL to pre-DBS levels [39]. This adjustment of expectations may be determined by a change in the psychosocial context, interactions with healthcare teams, and individual perceptions [39].

Collectively, these findings suggest the possibility of improving patient satisfaction and clinical outcomes by managing pre-operative expectations. Candidate patients for DBS should be selected using a multidisciplinary approach that provides comprehensive information. The clinical team should explain to the patients what they can realistically expect from the surgery, as well as the potential surgical complications and side effects [50]. The studies also highlighted that few tools have been validated to objectively assess the long-term expectations of patients with PD regarding DBS. To quantify these expectations, the reviewed studies used heterogeneous and invalidated instruments; therefore, they did not always allow a comparison of the results. Specifically, they administered semi-structured interviews [35], ad hoc questionnaires [37, 38, 40], and modified validated scales [36, 39]. For example, the PDQ-39 has been adapted to detect the degree of expectancy of improvement for each functional domain investigated using the instrument [36, 39]. However, this tool fails to reflect real changes, specifically in the psychosocial domains [36].

### Limitations

There are several limitations in this study, so our results should be interpreted with caution. First, although we used a standardized procedure for the revision process, we selected articles from a single database (PubMed) and in English only; therefore, we cannot exclude the possibility that other studies may have returned different results. In addition, the application of restrictive MESH terms led to a small number of results, so our review is certainly limited. Second, we analysed the therapeutic expectations specifically in patients with PD undergoing DBS, but not in those who were candidates and did not undergo surgical treatment. In the future, expanding the study population could enhance the data collected. Third, some studies had small normative samples, therefore, they may not have been sufficiently representative. Finally, but no less relevant, the high heterogeneity that characterized the experimental design and the nature of the outcome measures of the studies we included did not allow us to quantitatively summarize the results using meta-analytical approaches. Although this fact limits the generalizability of our positions, we still believe that a narrative approach to presenting and discussing the results of the included studies is still appropriate to the topic at hand.

## Conclusion

In conclusion, the data summarised in this review suggest the need for routine clinical practice to investigate and optimise patient expectations. Realistic and positive expectations can improve the efficacy of DBS, patient-perceived satisfaction, and subjective well-being. Patient education on DBS and multidisciplinary healthcare team-patient interaction play key roles in this process. However, the following gaps are emerging and need to be filled to improve the quality of care: the identification and use of validated and standardised tools for the assessment of treatment expectations, specifically in PD, and the implementation of standardised patient education protocols for DBS to meet the specific needs of individual patients.

## Data Availability

Not applicable.
